# Modeling dioxygenase enzyme kinetics in familial paraganglioma

**DOI:** 10.1242/bio.013623

**Published:** 2015-09-14

**Authors:** Justin P. Peters, Yeng F. Her, L. James Maher

**Affiliations:** Department of Biochemistry and Molecular Biology, Mayo Clinic College of Medicine, 200 First St. SW, Rochester, MN 55905, USA

**Keywords:** Paraganglioma, Hypoxia, HIF, PHD, Mathematical model

## Abstract

Hypoxia inducible factors (HIFs) play vital roles in cellular maintenance of oxygen homeostasis. These transcription factors are responsible for the expression of genes involved in angiogenesis, metabolism, and cell proliferation. Here, we generate a detailed mathematical model for the enzyme kinetics of α-ketoglutarate-dependent HIF prolyl 4-hydroxylase domain (PHD) dioxygenases to simulate our *in vitro* data showing synergistic PHD inhibition by succinate and hypoxia in experimental models of succinate dehydrogenase loss, which phenocopy familial paraganglioma. Our mathematical model confirms the inhibitory synergy of succinate and hypoxia under physiologically-relevant conditions. In agreement with our experimental data, the model predicts that HIF1α is not stabilized under atmospheric oxygen concentrations, as observed. Further, the model confirms that addition of α-ketoglutarate can reverse PHD inhibition by succinate and hypoxia in SDH-deficient cells.

## INTRODUCTION

Maintaining oxygen homeostasis is vital to many cellular processes. When a cell is deprived of oxygen, ATP production drastically decreases from 36 to 2 ATP molecules per molecule of glucose metabolized. To sense and adapt to a low oxygen environment, mammalian cells rely on two key mediators, HIF1α and HIF2α (hypoxic-inducible factors; referred to as HIFα) (Pugh and Ratcliffe, 2003; Wenger, 2002). HIFs are transcription factors with an oxygen sensitive alpha-subunit (HIFα) and a constitutively expressed beta-subunit (HIFβ). In normoxia, HIFα is constitutively hydroxylated by prolyl 4-hydroxylase domain (PHD) protein, which mediates HIFα interaction with the von Hippel-Lindau (VHL) E3 ubiquitin ligase complex that marks the transcription factor for rapid proteasomal degradation. During hypoxia, the enzymatic rate of PHD decreases allowing HIFα to remain stable and translocate to the nucleus, where it can dimerize with HIFβ to form transcriptionally active HIF complex. The HIF complex activates genes involved in glucose uptake, energy metabolism, angiogenesis, erythropoiesis, cell proliferation and apoptosis (Pugh and Ratcliffe, 2003; Wenger, 2002).

**Fig. 1.**
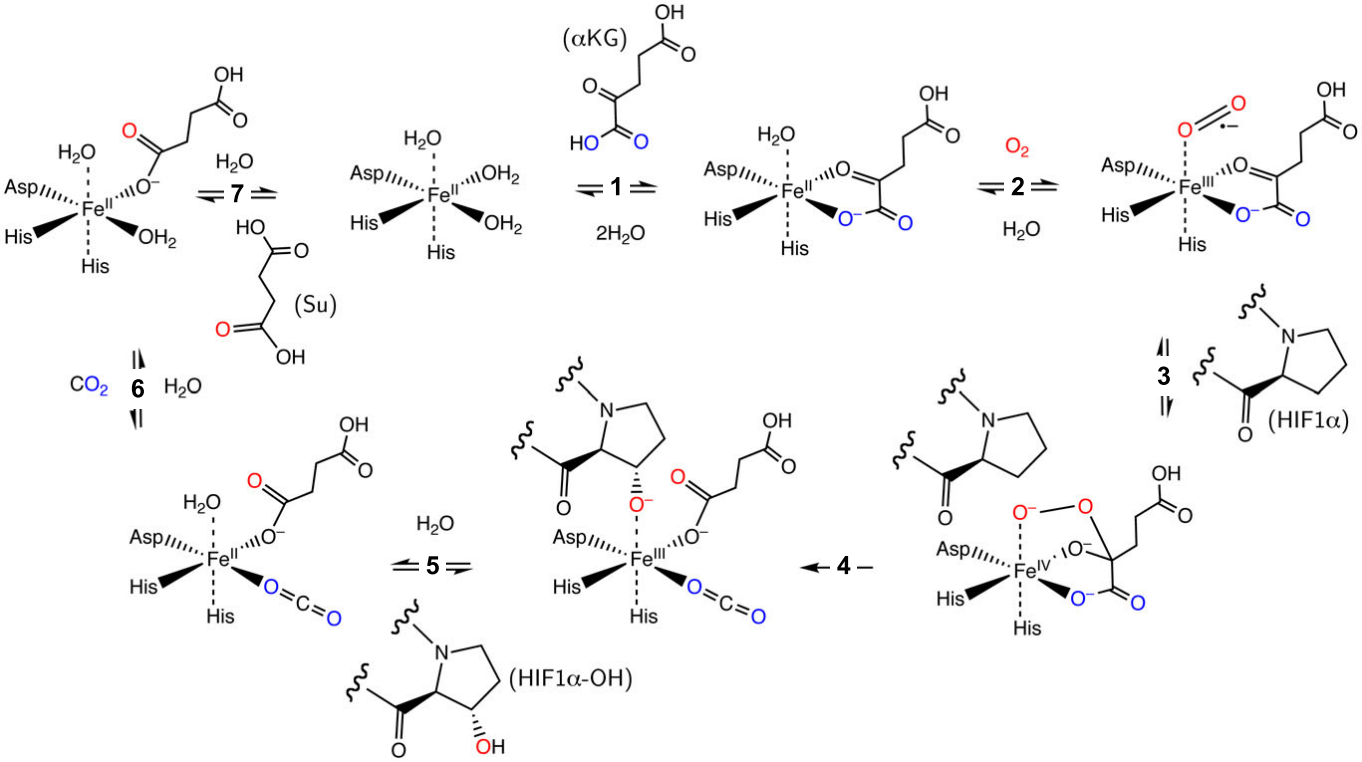
**Schematic representation of the ordered tri-tri PHD reaction mechanism (****Myllyla et al., 1977****).** PHD is assumed to be always bound by iron. PHD reacts with (1) α-ketoglutarate (αKG), (2) oxygen (O_2_), and (3) HIF-1α, sequentially, to form an Fe(IV) peroxyhemiketal bicyclic complex (Costas et al., 2004). The activated oxygen is cleaved resulting in the hydroxylation of HIF1α and decarboxylation of αKG (4). Hydroxylated HIF1α is released (5) then carbon dioxide (6) and succinate (7).

Mammalian cells express three PHD isoforms: PHD1, PHD2, and PHD3 (Berra et al., 2003). PHD1 is found exclusively in the cytosol. PHD2 is located in both the cytosol and nucleus, whereas PHD3 is only in the nucleus. PHD2 is believed to be the main oxygen sensor determining HIFα levels in a number of cell types. In fact, intracellular levels of PHD1 and PHD3 are almost undetectable in normoxia (Appelhoff et al., 2004). PHD enzymes operate in an ordered tri-tri reaction mechanism, requiring iron as a cofactor, α-ketoglutarate (αKG), and oxygen to hydroxylate HIFα, and producing carbon dioxide and succinate as by-products (Fig. 1) (Myllyla et al., 1977). In this mechanism, a 2-histidine-1-carboxylate facial triad (Hegg and Que, 1997) anchors the iron atom in the active site and maintains three coordination sites available for the substrates (Costas et al., 2004), which is consistent with the crystal structure of PHD2 (McDonough et al., 2006). Under conditions of reduced iron, αKG, or oxygen, the rate of PHD catalysis is severely reduced. PHD enzymes are also subject to competitive inhibition by Krebs cycle metabolites such as fumarate (Isaacs et al., 2005; Sudarshan et al., 2009; Xiao et al., 2012) and succinate (Letouze et al., 2013; Selak et al., 2005; Xiao et al., 2012) and by 2-hydroxyglutarate (Zhao et al., 2009) because of the structural similarity to αKG.

To date, a number of diseases have been hypothesized to involve PHD inhibition and HIFα activation (Pollard et al., 2005; Selak et al., 2005; Zhao et al., 2009). Here we focus on loss of succinate dehydrogenase (SDH) as it relates to the development of familial paraganglioma (PGL). In PGL, SDH loss leads to succinate accumulation in the mitochondria. Succinate can diffuse into the cytoplasm and competitively inhibit PHD resulting in the stabilization of HIFα (Selak et al., 2005).

**Fig. 2.**
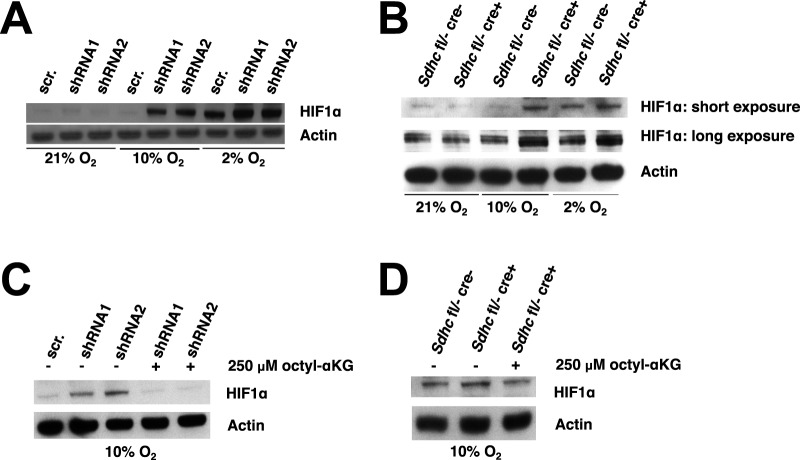
**Succinate accumulation and hypoxia synergistically inhibit PHD function in SDH deficient cells.** Data reproduced and figure modified from Fig. 2 of Her et al. (2015). (A) HEK293 cells were transduced with SDHB silencing lentiviruses (shRNA1 or shRNA2) or control vector (scrambled; scr.). After incubating cells in 21%, 10%, or 2% oxygen for 48 h, total cell lysates were subjected to western blot analysis to determine HIF1α levels. Actin serves as loading control. (B) Immortalized mouse embryonic fibroblasts (iMEFs) with the indicated genotypes were treated with 1 μM tamoxifen for 7 days and then incubated in 21%, 10%, or 2% oxygen for 48 h. Total cell lysates were subjected to western blot analysis to determine HIF1α levels, with actin serving as a loading control. (C) After incubation in 10% oxygen for 48 h, *SDHB* knockdown HEK293 cells were incubated with or without 250 μM octyl-αKG for 12 h. HIF1α levels were assessed by western blot analysis, with actin serving as a loading control. (D) Following treatment with 1 μM tamoxifen for 7 days then incubation in 10% oxygen for 48 h, *Sdhc* knockout iMEFs were incubated with or without 250 μM octyl-αKG for 12 h. HIF1α levels were assessed by western blot analysis. Actin serves as loading control.

While several mathematical models have been developed since 2004 to describe HIF signaling (Cavadas et al., 2013), most of the previous models are designed around the HIF pathway (Dayan et al., 2009; Kooner et al., 2006; Nguyen et al., 2013; Schmierer et al., 2010; Yucel and Kurnaz, 2007) – including at a minimum HIFα, PHD, VHL, and HIF-responsive elements (HRE) but also incorporating a combination of HIFβ, factor inhibiting HIF (FIH), vascular endothelial growth factor (VEGF), arrest-defective protein (ARD), and the transactivation domains of HIFα (N-TAD and C-TAD) – or the entire hypoxia response control network (Kohn et al., 2004; Yucel and Kurnaz, 2007). At a pathway or network level these models ignore the roles of α-ketoglutarate and succinate. One previous model did explicitly incorporate all of the PHD reactants (Qutub and Popel, 2006) but did not include the product succinate until its subsequent expansion (Qutub and Popel, 2007). In this expanded model, HIFα was completely stabilized at 21% oxygen independent of the presence of accumulated succinate. This prediction is not consistent with our observations in SDH deficient cells (Her et al., 2015). We recently developed a human embryonic kidney HEK293 lentiviral shRNA SDHB knockdown cell culture model where no detectable HIFα stabilization was observed upon succinate accumulation in 21% oxygen (Fig. 2A). Only when oxygen was lowered to 10% or 2% was HIFα stabilized. We also developed an immortalized mouse embryonic fibroblast *Sdhc* knockout model, which shows minimal HIFα stabilization at 21% oxygen and overall decreased HIFα responsiveness (Fig. 2B). From these cell culture models, we deduced that succinate accumulation and hypoxia synergistically inhibit PHD and concluded that an improved mathematical model was needed.

Can *in silico* tools be generated that accurately predict HIF levels at both physiologically-relevant as well as atmospheric oxygen conditions? Can this model capture the effects of succinate accumulation on PHD activity? Can this initial framework be expanded to more fully describe the HIF network, i.e. additional isoforms, greater crosstalk, and more compartments? And once developed, will this model make meaningful predictions, such as αKG and hyperoxia levels required for therapeutic treatment of PGL? These questions are the driving force for this work. Using our *in vitro* data as motivation and founded on PHD enzyme kinetics we developed an ordinary differential equations-based model to provide a quantitative framework for understanding HIF1α hydroxylation mediated by PHD2 and a plausible mechanistic explanation for the synergistic effect of succinate and hypoxia. We show that this model predicts that succinate and hypoxia synergistically inhibit PHD, and this inhibition can be overcome by the addition of αKG. Simulations from our model suggest new testable hypotheses, which can guide future experiments in a rational way, and this work further reinforces the notion that αKG and/or hyperoxia could be therapeutic in PGL.

## RESULTS

### Model assumptions and limitations

The challenges in modeling the HIF pathway are as follows: there are three PHD isoforms and each may differentially contribute to HIF regulation. There exists a compartmentalization of these proteins and other factors in the HIF response, and specific localizations within the cell can affect rates and activities. Additionally, three isoforms of HIFα exist and each can regulate a specific set of genes. Thus, mathematical models face enormous obstacles in trying to account for all of the possible signaling crosstalk and to account for the abundance (and potential modified state) of each of the players in all of their potential subcellular locations. Moreover, there exists feedback regulation within the HIF pathway as well as crosstalk and synergism with other pathways, e.g. NFκB and mTOR (Cavadas et al., 2013). Consequently, several assumptions must be imposed due to a lack of experimental data and to simplify the modeling process.

Our model incorporates key molecular interactions in the HIF1α hydroxylation process mediated by PHD2. The molecular components and steps of the hydroxylation reaction are presented in Fig. 1. Detailed discussion of the model including reactions, reaction rates, and parameters are described in Materials and Methods. Under normoxic conditions, HIF1α protein is produced at a steady rate, but the protein is degraded either by non-specific protein turnover or from oxygen-sensitive hydroxylation and the resulting von Hippel-Lindau (VHL)-mediated proteasomal degradation. In this initial stage of model development, we omit any molecular details of the VHL complex and we assume that once hydroxylated, HIF1α is committed towards a proteasomal destruction fate. This is accomplished by setting to zero the rate constant for the one term influenced by hydroxylated HIF1α (see Materials and Methods). Future iterations of the model will incorporate the VHL pathway in greater detail.

Although the PHD isoforms have specific cytoplasmic and/or nuclear localizations, for simplicity in this initial development of the model we have considered only a single PHD entity – PHD2. Furthermore, since the levels of the various reactants and products (and their fluxes) are not specifically known in the mitochondria, the nucleus, and the cytoplasm, we assume a single cellular compartment as opposed to a multi-compartment model. Conceptually, this is equivalent to a well-mixed cytosolic compartment where metabolites like αKG and succinate can freely move in and out of the mitochondria and PHD2 can freely translocate in and out of the nucleus. In such a model, changes to these global levels can reflect different subcellular or environmental contexts. Finally, we have focused our attention on only the HIF1α isoform at present, leaving the incorporation of other isoforms for future iterations.

### Succinate and hypoxia inhibition of PHD2

**Fig. 3.**
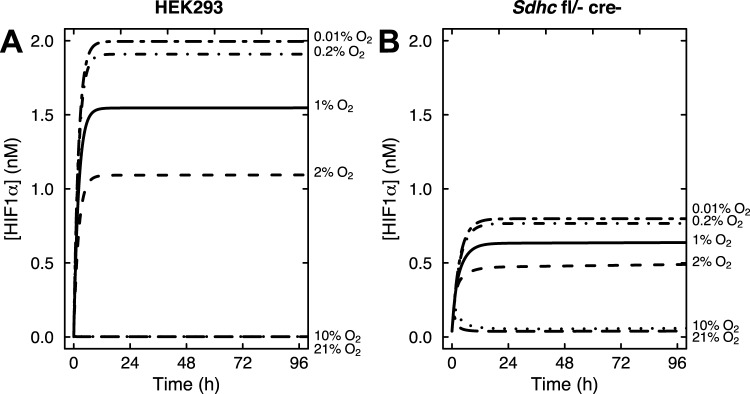
**Mathematical model results showing the time course of HIF1α accumulation at oxygen levels ranging from 0.01% to 21% oxygen, as indicated.** (A) Control *SDHB* knockdown HEK293 cells. (B) Control *Sdhc* knockout fibroblasts.

**Fig. 4.**
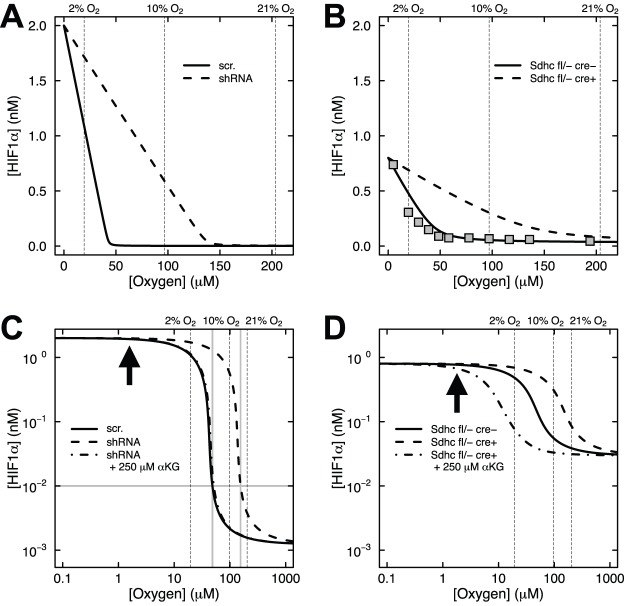
**Model predictions of the effects of succinate accumulation and hypoxia on HIF1α concentration under endogenous αKG conditions or αKG rescue.** (A) *SDHB* knockdown HEK293 cells transduced with scrambled (scr.) shRNA control or *SDHB* knockdown shRNA (shRNA) driving succinate accumulation (solid and dashed lines, respectively). (B) *Sdhc* knockout fibroblasts with control genotype (*Sdhc* fl/− cre−) or genotype driving succinate accumulation (*Sdhc* fl/− cre+). Data from HeLa cells (filled squares) are shown for comparison (Jiang et al., 1996). (C,D) Log-log plots of A and B showing the effects of succinate accumulation and hypoxia on HIF1α concentration under endogenous αKG conditions or with 250 μM αKG treatment (dot-dash lines). Bold arrows indicated 0.2% oxygen. Gray lines in C show that 0.01 nM HIF1α corresponds to 5% oxygen in control cells, but 16% oxygen in *SDHB* knockdown cells.

Our *in vitro* experiments (Her et al., 2015) showed that HIF1α is stabilized at 2% oxygen in control cells and at both 10% and 2% oxygen upon succinate accumulation due to loss of SDH function (Fig. 2A,B). Stabilization of HIF1α was not observed at 21% oxygen with or without succinate accumulation. We first applied our mathematical model to simulate the synergistic inhibition of PHD by succinate and hypoxia, using the intracellular concentrations of αKG and succinate measured in our SDH-loss cell culture experiments (Table 1). Succinate is a competitive inhibitor of PHD2 in addition to being a product of the reaction (Myllyla et al., 1977). Fig. 3 shows HIF1α concentration as a function of time using measured levels of αKG and succinate determined in untreated cells (Table 1) and selected oxygen amounts ranging from 0.01% (∼anoxia) to 21% (atmospheric). The model predicts that under hypoxic conditions, HIF1α protein is rapidly stabilized, reaching steady-state levels within ∼12 h (the HIF1α concentration achieved being inversely related to oxygen amount). At steady state, PHD2 activity balances the level of HIF1α being synthesized with the level of HIF1α being degraded. Fig. 4 shows the dependence of HIF1α concentration on the combined effects of PHD inhibition by succinate and hypoxia oxygen levels simulated at 48 h post exposure to hypoxia. This time point mimics our cell culture experiments (Fig. 2A,B). At 21% oxygen, both non-specific target (scr.) and shRNA SDHB target (shRNA) lentiviral-treated HEK293 cells did not stabilize HIF1α (Figs 2A and 4A). At 10% oxygen, we observed a dramatic increase in HIF1α levels (shRNA versus scr.), and at 2% oxygen HIF1α is stabilized under both conditions. Our mathematical model qualitatively confirms this observation (Fig. 4A). Similarly in the knockout model, *Sdhc* fl/− cre− control cells only exhibit appreciable HIF1α stabilization at 2% oxygen, whereas *Shhc* fl/− cre+ cells have stabilized HIF1α at both 10% and 2% oxygen (Fig. 4B). For comparison, data from HeLa cells (Jiang et al., 1996) are plotted along with the results in Fig. 4B. While cell-type specific differences undoubtedly exist, it is remarkable how well the model agrees with the experimental data from another cell type. However, the HeLa cell data suggest an even stronger dependence on oxygen levels, showing a majority of HIF1α degraded already at 2% oxygen.

**Table 1.**
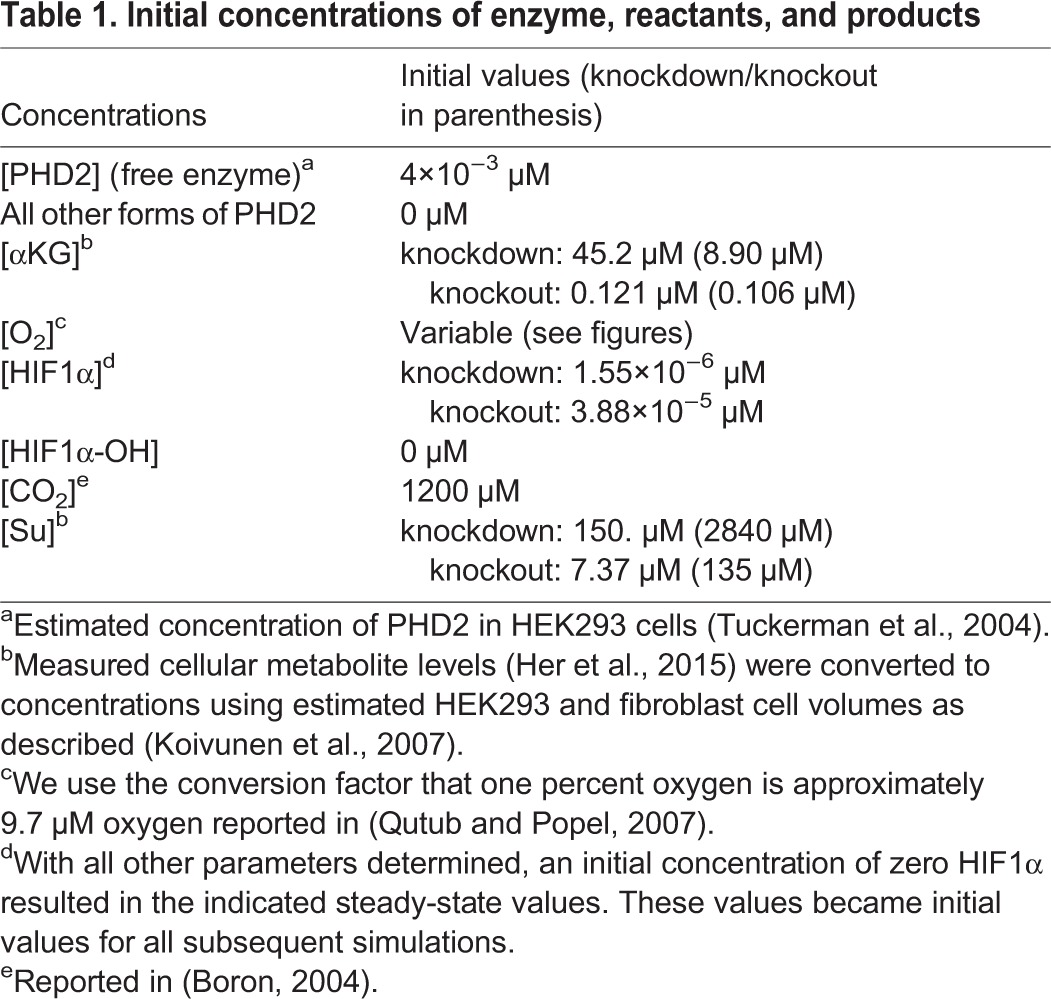
**Initial concentrations of enzyme, reactants, and products**

The dependence of HIF1α concentration on oxygen levels is more fully appreciated in log-log plots (Fig. 4C,D). There is marked sensitivity in the range of 2% to 10% oxygen for control cells, and this shifts to 10% to 21% upon succinate accumulation in knockdown or knockout cells. HIF1α appears maximally stabilized at 0.2% oxygen (bold arrows, Fig. 4C,D), consistent with experimental data from HeLa cells where knockdown of both PHD2 and PHD3 in these cells did not lead to much further HIF1α stabilization (Fig. 5B of Stiehl et al., 2006). The model can be utilized to make predictions about the therapeutic rescue of hyperoxia. Consider 5% oxygen in control HEK293 cells, where the concentration of HIF1α is 0.01 nM (Fig. 4C). In order to achieve this concentration of HIF1α in SDH-loss cells, the model predicts that the oxygen level would need to be increased to 16% (gray lines, Fig. 4C). Thus, our enzyme kinetic model confirms our previous experimental result (Her et al., 2015) that PHD is synergistically inhibited by succinate accumulation and hypoxia in cell culture SDH-loss models of PGL.

### αKG overcomes PHD inhibition by succinate accumulation and hypoxia

As originally shown by others (MacKenzie et al., 2007; Smith et al., 2007), we also showed in our SDH-loss models of PGL that addition of cell-permeable αKG could overcome succinate inhibition of PHD (Her et al., 2015). This is rationalized by the fact that succinate and αKG compete for the active site of PHD2 (Letouze et al., 2013; MacKenzie et al., 2007; Xiao et al., 2012). In our SDH-loss cell culture experiments, we observed that addition of 250 μM cell-permeable octyl-αKG reversed the synergistic inhibition of PHD by succinate and hypoxia (10% oxygen) in both knockdown and knockout cells (Fig. 2C,D). To model this effect, we mimicked the cell culture experiments and simulated a 48-h incubation at 10% oxygen before adding 250 μM αKG for an additional 12 h. The results demonstrate that addition of 250 μM αKG completely reverses the combined succinate and hypoxia inhibition of PHD2 to produce HIF1α concentrations observed in the control cells (dot-dash line, Fig. 4C). In fact, the model prediction for the knockout cells is an over-rescue at this αKG concentration (dot-dash line, Fig. 4D). This result further validates our mathematical model, showing that it qualitatively confirms the results of our *in vitro* experiments showing αKG reversal of PHD inhibition by succinate and hypoxia.

### Quantitative effect of hypoxia on HIF1α and PHD2 synthesis

HIF1α protein concentration in hypoxia is dependent on the expression and enzymatic activity of PHD (Stiehl et al., 2006). PHD mRNA levels are reportedly upregulated at 4 h post hypoxia (Cioffi et al., 2003), and PHD increases in concentration by three-fold over 48 h following exposure to hypoxia (1% oxygen) (Stiehl et al., 2006). Specifically, upon exposure to hypoxia (1% oxygen), it has been observed that HIF1α is stabilized, reaching a maximal concentration after 4 to 8 h. HIF1α protein concentration then decreases as PHD expression is increased, reaching a steady state after 48 h (Stiehl et al., 2006). We therefore introduced into our model a pulse of new PHD2 (in the free enzyme form) that is proportional to HIF1α concentration to reproduce the HIF1α and PHD2 experimental data (see Materials and Methods):
(1)
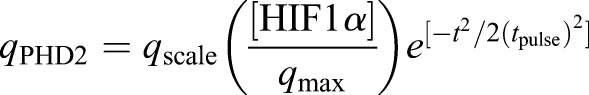



This pulse takes the form of a Gaussian distribution (centered at 0 min with width corresponding to a standard deviation of *t*_pulse_), and is proportional to HIF1α concentration normalized by *q*_max_ and scaled by *q*_scale_ so that a 3-fold increase in PHD2 (total concentration of all forms of the enzyme) is observed at 1% oxygen, consistent with the experimental data:
(2)
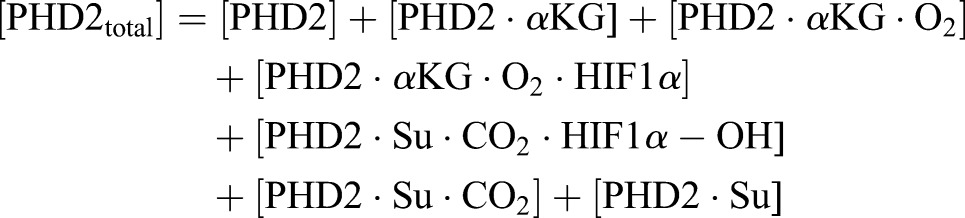

**Fig. 5.**
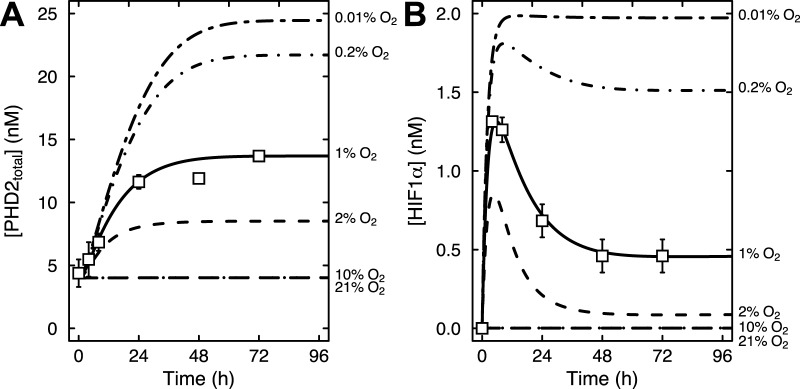
**Predictions of the expanded model including feedback regulation.** Results of the model with feedback regulation added showing the time course of PHD2 (A) and HIF1α (B) levels at oxygen amounts ranging from 0.01% to 21% oxygen, as indicated. Reported experimental data from HEK293 cells (Stiehl et al., 2006) were used to calibrate the model and are shown as open symbols.

Fig. 5 illustrates predicted PHD2 and HIF1α concentrations as a function of time from this expanded model including feedback for oxygen conditions ranging from 0.01% to 21%, along with the experimental data for HEK293 cells exposed to 1% oxygen for 72 h reported in (Stiehl et al., 2006). The dependence of PHD2 concentrations on oxygen in Fig. 5A shows that between 10% and 21% oxygen, PHD2 levels do not appear to be affected by oxygen concentration. However, at 2% oxygen (or less), PHD2 levels increase at least two-fold (and up to ∼6-fold) before reaching a steady state at 48 h post hypoxia. Additionally, HIF1α protein levels are predicted to be dependent on oxygen and increasing PHD2 concentrations, as expected (Fig. 5B). Between 10% and 21% oxygen, HIF1α is rapidly degraded by PHD2. In contrast, from 2% to 0.2% oxygen, HIF1α levels quickly increase before sufficient PHD2 is synthesized to compensate partially. Steady state is then reached at a lower HIF1α level than observed previously (Fig. 3A), and occurs immediately following the steady state in PHD2 concentration (∼48 h). Below 0.2% oxygen, HIF1α levels are predicted to be unaffected by increasing PHD2 levels, as oxygen is unavailable to hydroxylate HIF1α (Fig. 5B). More recent studies in HEK293 cells confirm a maximum in HIF1α stabilization following 8 h at 1% oxygen, whereas this effect is drastically reduced at 3% oxygen (Nguyen et al., 2013), consistent with our model.

### Adaptation of the HIF system to respond to subsequent hypoxic insults

**Fig. 6.**
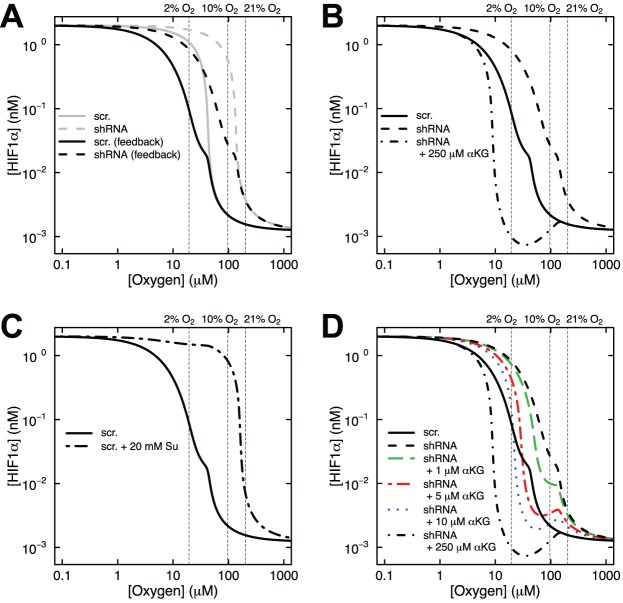
**Model results exploring the interplay of αKG and succinate concentrations in controlling HIF1α levels at various oxygen amounts.** (A) Predictions of the model with or without added feedback regulation showing the effects of succinate accumulation and hypoxia on HIF1α concentration under endogenous αKG conditions. See legend of Fig. 4 for details. (B) Results of αKG rescue of PHD2 function from synergistic inhibition by succinate and hypoxia when feedback regulation is included in the model. See legend of Fig. 4 for details. (C) Model with feedback predictions for control *SDHB* knockdown HEK293 cells incubated with or without 20 mM succinate (Su) for 12 h. (D) Revisiting the predictions of B, *SDHB* knockdown HEK293 cells were treated with 1 μM αKG (green, long dash line), 5 μM αKG (red, dot-dash line), or 10 μM αKG (blue, dotted line).

After shifting to hypoxic conditions, HIF1α is stabilized and initiates hypoxic signaling. A negative feedback response partially decreases HIF1α concentration through an increase in PHD2. It has been proposed that the feedback control of HIF1α creates a new set point in the system, and allows room for a future increase in HIF1α stabilization in response to a further hypoxic shift (Stiehl et al., 2006). HEK293 cells shifted to 1% oxygen for 72 h responded to a shift to 0.2% oxygen with increased stabilization of HIF1α (Stiehl et al., 2006). This response was also followed by a resetting of stabilized HIF1α levels over the course of 4 h, presumably in preparation for a subsequent insult. Our model nicely illustrates this concept (Fig. 6A). Without feedback regulation (gray curves, Fig. 6A), HIF1α concentrations approach the theoretical maximum. Since oxygen levels are already low, the production of additional enzyme is necessary so that the system is once again poised to respond to any further decrease in oxygen. This results in a resetting of HIF1α levels below a certain oxygen threshold (black curves, Fig. 6A). The observed feedback regulation has important therapeutic implications. When we model our cell-permeable αKG experiment in the context of a feedback system, we see that the model predicts an over-rescue of the cells with 250 μM αKG (Fig. 6B), reminiscent to that predicted in the knockout cells (Fig. 4D). This result suggests that negative feedback regulation of HIF1α will make cells more sensitive to αKG therapy.

### HIF1α levels are determined by the ratio of αKG to succinate

The experimental values for intracellular succinate and αKG concentrations shown in Table 1 demonstrate that succinate accumulates in SDH-loss cell culture models, as expected. Interestingly, there is also a concomitant decrease in αKG concentration. These reciprocal changes exacerbate the observed ratio of αKG to succinate. To explore this idea, we used our expanded mathematical model with feedback regulation to simulate the effect of addition of cell-permeable succinate to control HEK293 cells. Experimental data at 10% and 21% oxygen with addition of 20 mM dimethyl succinate show increased HIF1α stabilization only at 10% oxygen (supplementary material Fig. S4C of Her et al., 2015), though it is difficult to determine how much of the added compound enters cells and is de-esterified. Again our simulations mimic the experimental conditions with a 48-h incubation in hypoxia before adding 20 mM succinate for an additional 12 h. The model predicts a dramatic stabilization of HIF1α at 10% oxygen but not at 21% oxygen, even with this high concentration of added succinate (Fig. 6C), consistent with the experimental results. The model suggests that if this high level of succinate accumulation were achieved, more αKG would be needed to counter the effect. Thus, the balance of αKG to succinate is a key determinant of HIF1α levels.

Lastly, we used our model to predict that the ability of αKG to overcome the effects of succinate accumulation in HEK293 cells. The model predicts that small changes in αKG concentration (relative to succinate), should have dramatic effects on HIF1α concentration (colored curves in Fig. 6D). Addition of 10 μM αKG restores HIF1α concentration to the level of control cells at both 2% and 10% oxygen (blue dotted line, Fig. 6D). The addition of 5 μM αKG is likewise predicted to have large effects (red dot-dash line, Fig. 6D), and even addition of 1 μM αKG may have therapeutic effect when combined with hyperoxia. Future studies will subject these predictions to experimental validation.

## DISCUSSION

Motivated by our *in vitro* experimental studies, we here utilize first principles of enzyme kinetics to generate a mathematical model (with or without feedback regulation) that can rationalize the observed synergistic inhibition of PHD by succinate and hypoxia in an SDH-loss cell culture model of PGL. Using experimental values for intracellular succinate and αKG concentrations (Her et al., 2015), we showed that this model confirms the observed synergistic inhibition of PHD by succinate and hypoxia and also showed how this inhibition can be overcome by addition of αKG. Of particular interest is the predicted range of PHD inhibition by succinate and hypoxia. As shown with our model, SDH-loss cells cultured in 21% oxygen do not stabilize HIF1α because PHD quickly hydroxylates HIF1α and the protein is rapidly degraded (Fig. 4). However, in the physiological oxygen range, our model predicts that HIF1α is stabilized by the combined inhibition of succinate and hypoxia (Fig. 4). The model results are consistent with experimental data and demonstrate how atmospheric oxygen level masks important biology that can be observed under physiologically-relevant conditions.

The next step in the development of this mathematical model will be to predict other experimental results that can then be validated by related experimental systems in our laboratory. It is likely that the ratio of PHD2 to HIF in different tissues and cell types will likely modulate the extent of HIF accumulation, and this avenue will be explored in the future. Additionally, simulations of acute hypoxia versus chronic will be used to refine the model moving forward. Relevant to the SDH-loss model of PGL, predictions of αKG and hyperoxia levels required for therapeutic treatment of PGL are an important avenue of future study. Our model can also predict effects of succinate accumulation and hypoxia on HIF2α levels. Additionally, other PHD isoforms can be readily incorporated into the model. More experiments will need to be performed to determine if the model confirms western blot data for HIF2α and HIF3α as well as PHD1 and PHD3 obtained in such cell culture models. While the model currently uses values from shRNA SDHB knockdown cells and *Sdhc* knockout cells (Her et al., 2015), future simulations with parameters more relevant to neuroendocrine cells will be performed. Additionally, iron will be explicitly incorporated into the model, allowing for simulations of competitive inhibitors such as cobalt or iron chelators such as deferoxamine. In summary, this mathematical model framework for the PHD dioxygenases can be applied to gain insight into effects of iron, succinate, hypoxia, and αKG on PHD and HIFα, guiding future experimental designs.

## MATERIALS AND METHODS

### The kinetic model


(3)
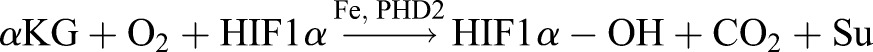

Eqn 3 describes the overall chemical reaction catalyzed by PHD. Succinate, a by-product of the reaction, is structurally similar to αKG, a reactant, and can compete with αKG for interaction with the active site of PHD2 (Myllyla et al., 1977; Tuderman et al., 1977). In SDH-deficient models, succinate accumulates and stabilizes HIF1α by inhibiting PHD2 (Letouze et al., 2013; MacKenzie et al., 2007; Selak et al., 2005; Xiao et al., 2012).

In our mathematical model, we assume that PHD2 is always bound to iron. Iron binding can explicitly be introduced into the model in the future. We also assume reversible binding of PHD2 to both the reactants and products: αKG, oxygen, HIF1α, hydroxylated HIF1α, carbon dioxide, and succinate. The ordered tri-tri mechanism of PHD2 (Fig. 1) is then captured in Eqns 4 through 10.
(4)
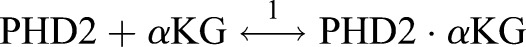

(5)
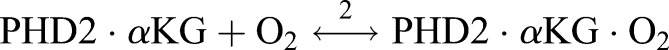

(6)
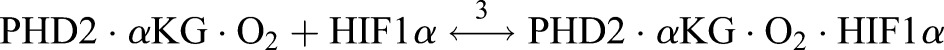

(7)
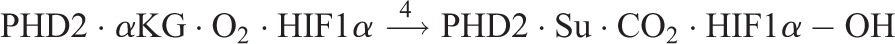

(8)
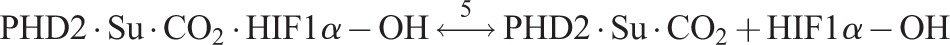

(9)
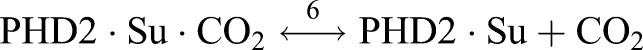

(10)
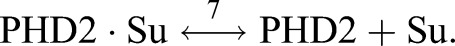



The catalytic step of the reaction (shown in Eqn 7) is highly exothermic, so we consider it irreversible. In addition, we assume that HIF1α is produced at a constant rate, and that oxygen-independent degradation of HIF1α (turnover) follows first-order kinetics:
(11)
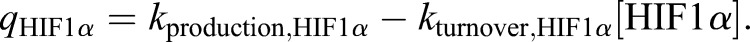



Using the principle of mass action, the ordinary differential equation (ODE) system governing the reaction dynamics is then as follows:
(12)
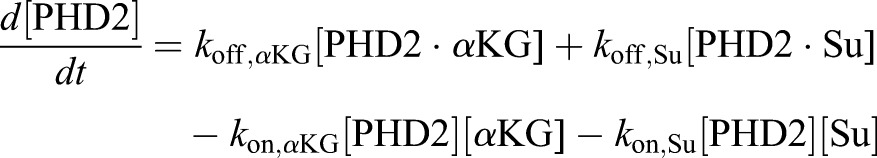

(13)
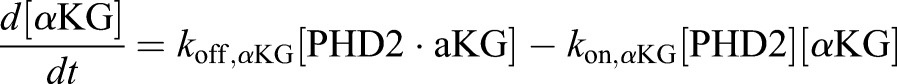

(14)
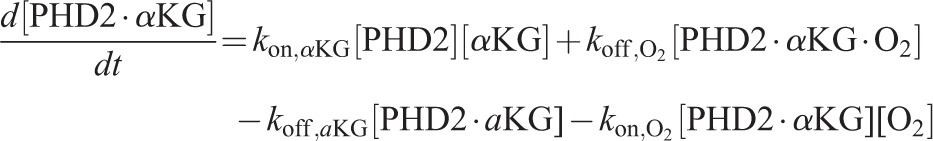

(15)
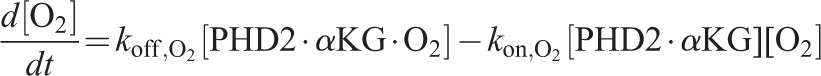

(16)
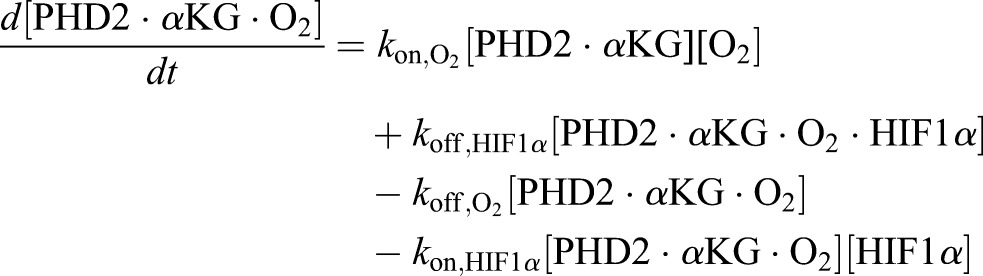

(17)
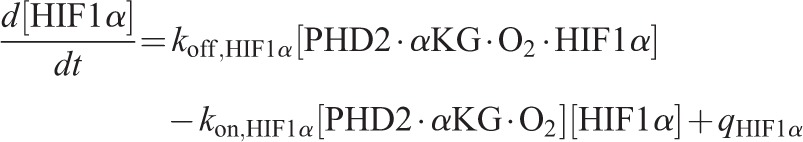

(18)
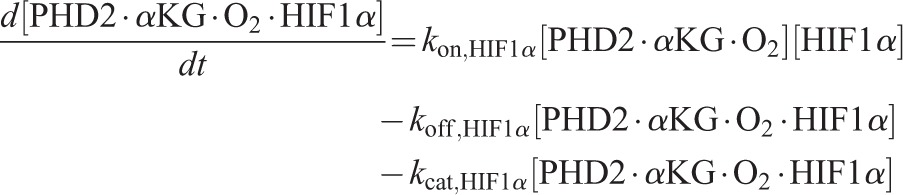

(19)
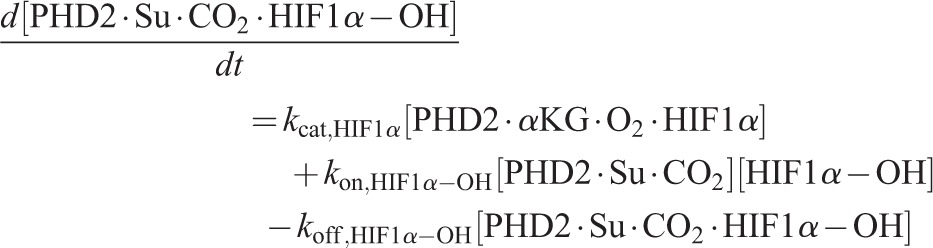

(20)
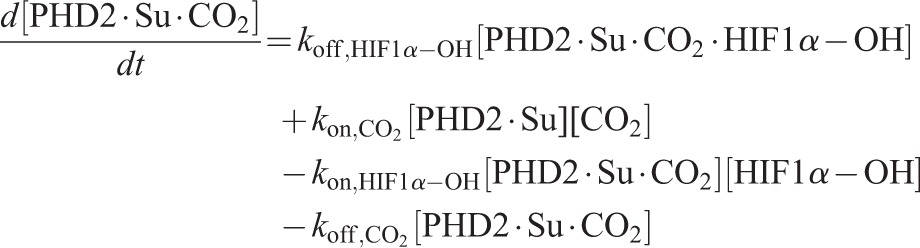

(21)
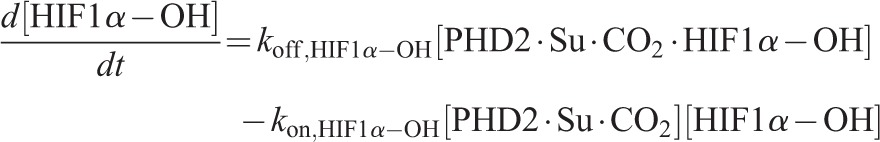

(22)
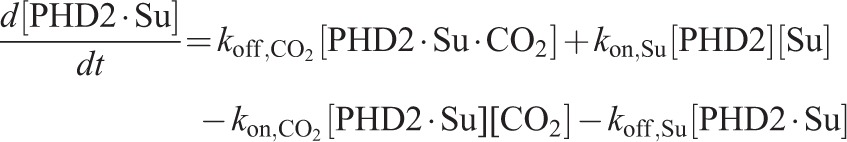

(23)
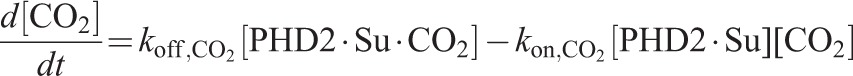

(24)
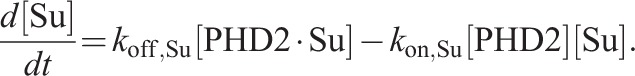

We make the assumption that as soon as HIF1α-OH is produced, it is quickly marked for proteasomal degradation by the VHL pathway. The simplest way to accomplish this in the model is to set *k*_on,HIF1α-OH_ to zero. In reality, HIF1α-OH is continuously accumulating in the model (because there is no VHL pathway). However, this accumulation is suppressed (has no effect) in the model if the rate *k*_on,HIF1α-OH_ is set to zero. We feel that this action is justified for two reasons. First, the step in question (the reverse reaction shown in Eqn 8) follows the irreversible catalytic step (shown in Eqn 7) and the reverse pathway has therefore reached a dead-end. Secondly, the rate in question is expected to be very small relative to the forward rate since the hydroxylated product is unlikely to re-bind the PHD2 active site once the Fe(IV) peroxyhemiketal bicyclic complex has broken apart but before other products are released. More detailed kinetics of VHL-mediated degradation will be introduced into the model in the future and *k*_on,HIF1α-OH_ will be allowed to attain non-zero values. Finally, we note that the steady-state concentration of HIF1α is given by setting Eqn 17 to zero and solving for HIF1α. In the limit of zero oxygen (i.e. eliminating every species containing oxygen), we have:
(25)
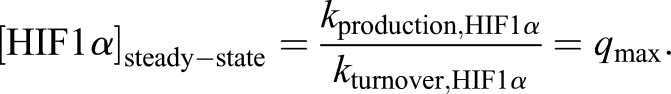

Therefore, the maximum theoretical concentration of HIF1α in the model at steady state (*q*_max_) is determined by the production and turnover rates of HIF1α. Importantly, the steady states achieved at 21% oxygen are stable for greater than 4 days (exceeding the length of a typical cell culture experiment reported here).

### Parameter estimation

The system of nonlinear differential equations was solved with R software (Version 2.13.0) using the lsoda ODE solver in the package deSolve (Soetaert et al., 2010). Initial concentrations of all species are given in Table 1. From the rate equations, there are 15 unknown rate parameters: *k*_on,αKG_, *k*_off,αKG_, *k*_on,O2_, *k*_off,O2_, *k*_on,HIF1α_, *k*_off,HIF1α_, *k*_cat,HIF1α_, *k*_on,HIF1α-OH_, *k*_off,HIF1α-OH_, *k*_on,CO2_, *k*_off,CO2_, *k*_on,Su_, *k*_off,Su_, *k*_production,HIF1α_, and *k*_turnover,HIF1α_. Estimates of these parameters were determined by nonlinear least squares fitting of the model to an estimation of the experimental data in Fig. 2A (see below) using the simplex and inductive search hybrid (SIH) algorithm (Offord and Bajzer, 2001). Qualitatively for the scr. control: at 21% and 10% oxygen levels HIF1α is essentially unchanged from its starting value but sharply rises at 2% oxygen approaching the theoretical maximum value *q*_max_. For the shRNA case: HIF1α is essentially unchanged from its starting value only at 21% oxygen and is substantially larger at 10% and nearly maximal at 2% oxygen. In practice, this was accomplished by least squares fitting to the vector of values (0,0,0.5,0,0.25,0.85)×*q*_max_. The fitted values of the rate constants are reported in Table 2.

**Table 2.**
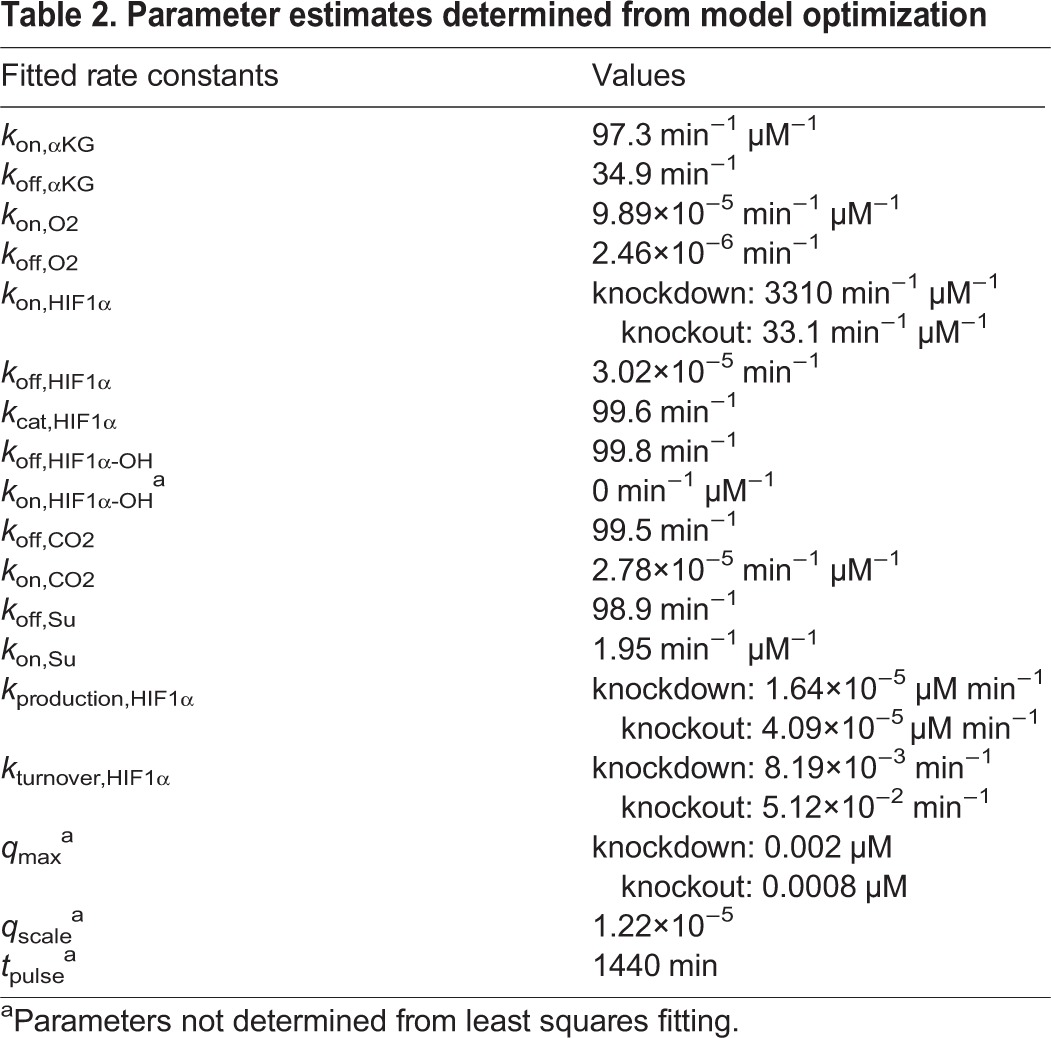
**Parameter estimates determined from model optimization**

Next, we reasoned from the qualitative data in Fig. 2 that the maximal level of HIF1α attained is decreased in the knockout cells as compared to the knockdown cells and that the basal level of HIF1α at 21% oxygen is slightly increased. Not having quantitative information about these differences, we arbitrarily adjusted the three model parameters most directly responsible for these effects by a scale factor: increased *k*_production,HIF1α_ by factor of 2.5, increased *k*_turnover,HIF1α_ by a factor of 6.25, and decreased *k*_on,HIF1α_ by a factor of 100 (Table 2).

The last step in model validation was to confirm that the parameter estimates are both plausible and reasonable and to determine if the kinetics can effectively be simplified in some instances. For example, the rate of CO_2_ re-entering the complex after catalysis is negligible (2.78×10^−5^ µM^−1^ min^−1^) as expected. This indeed does lead to a high *K*_d,CO2_ value (3.5 M), which is consistent with crude estimates of the *K*_i_ for CO_2_ on the order of at least mM (Myllyla et al., 1977). Additionally, while the value of *K*_d,HIF1α_ is low due to the small off rate, the value of *K*_m,HIF1α_ (3 µM) is consistent with reported values (Hirsila et al., 2003). Finally, it has been reported that the upper limit of a diffusion-limited rate could reach 10^10^ M^−1^ s^−1^ (or 6×10^5^ µM^−1^ min^−1^), so that *k*_on,HIF1α_ in the knockdown cells while large (3310 min^−1^ μM^−1^) has not reached this limit.
